# Detection and Quantification of Delamination Failures in Marine Composite Bulkheads via Vibration Energy Variations

**DOI:** 10.3390/s21082843

**Published:** 2021-04-17

**Authors:** Cristobal Garcia, Alfonso Jurado, Oscar Zaba, Publio Beltran

**Affiliations:** Técnicas y Servicios de Ingeniería S.L., Avda. Pio XII, 44, 28016 Madrid, Spain; alfonso.jurado@tsisl.es (A.J.); oscar.zaba@tsisl.es (O.Z.); publiobp@tsisl.es (P.B.)

**Keywords:** composite laminates, delamination, sensing systems, vibration-based monitoring, non-destructive evaluation, vibrations

## Abstract

This paper proposes a new vibration-based structural health monitoring method for the identification of delamination defects in composite bulkheads used in small-length fiber-based ships. The core of this work is to find out if the variations of vibration energy can be efficiently used as a key performance indicator for the detection and quantification of delamination defects in marine composite bulkheads. For this purpose, the changes of vibrational energy exerted by delamination defects in sandwich and monolithic composite panel bulkheads with different types of delamination phenomenon are investigated using a non-destructive test. Experiments show that the overall vibration energy of the bulkheads is directly dependent on the damage conditions of the specimens and therefore, the variations of this parameter are a good indicator of the incorporation of delamination defects in composite bulkheads. Additionally, the overall vibration energy changes also give interesting information about the severity of the delamination defect in the panels. Hence, this methodology based on vibratory energy can be used to accurately determine delamination defects in medium-sized composite bulkheads with the advantages of being a simple and cost-effective approach. The findings of this research possess important applications for the identification of delamination failures in composite components such as bulkheads, turbine blades, and aircraft structures, among others.

## 1. Introduction

Composite laminates have been extensively used in aerospace [1,2], marine [3,4], and automotive components [5] due to their high Young’s modulus and strength combined with low specific weight [6]. The weak point of these laminar materials is their poor interlaminar strength that can lead to critical delamination defects, and therefore significant losses of stiffness and strength in this type of laminar material [7]. Delamination failures can easily arise during the manufacturing process (e.g., drop tool impact, injection molding processing faults, incompatible materials blended together, etc.) or during the operation service of the component (e.g., bird strikes, hailstone impacts, matrix and shear cracks, etc.). In general, the detection of delamination defects is not a simple task, requiring sophisticated inspection methods for the detection of delamination breakages, which are not always visible from the outside [8].

In the last decade, a large number of non-destructive methods have been reported by the scientific and industrial community for the analysis of delamination defects in fiber-reinforced polymer (FRP) materials. Most of the reported papers suggested the techniques of ultrasounds [9,10], thermography [11], shearography [12], radiography [13], acoustic emission [14], and vibration-based solutions [15,16,17,18] for the detection and characterization of different defects in composite materials. Among the aforementioned techniques, vibration-based methods are considered as one of the most popular methods for damage detection because of their simplicity, versatility, and low cost, which facilitates their application to a more industrial environment.

The research focusing on how delamination defects influence the modal parameters of composite laminates started several decades ago and it has been widely reported by a large number of scientific studies [19,20,21,22,23]. The fundamental idea behind a modal analysis method is that the modal parameters (natural frequencies, damping, and vibration mode shapes) are functions of the physical properties of the composite structure (mass, damping, and stiffness). Therefore, changes in the physical properties of the material as per the example of stiffness reduction due to delamination breakages will cause changes in the modal characteristics.

With regards to the natural frequencies, a vast number of the publications published in this field reported that the presence of delamination defects in composite laminates produces small percentual changes in the natural frequencies [24,25,26]. An interesting contribution in this field was reported by [24]. The first ten natural frequencies of glass fiber epoxy beams were extracted for various case studies with different delamination characteristics. The findings of this paper revealed that the delamination defects have a slight effect on the natural frequencies of glass fiber epoxy laminates. However, the miniscule shift of the natural frequencies is within the range of precision of the experiments and, therefore, it is not sufficient for the detection of delamination. Other research studies [16,18] investigated the potential of the natural frequencies as a diagnostic tool for the detection and quantification of delamination defects. In general, it is known that the intensity of the natural frequency changes depends on the size of the delamination defect, and therefore, it is expected that larger delamination defects lead to higher decrements of the natural frequencies due to the greater loss of local stiffness.

In parallel, the potential of the vibratory mode shape changes as a key performance indicator (KPI) of the damage state of composite laminates was also examined in a large number of research works [27,28,29]. The above-mentioned studies [27,28] investigated the influence of delamination defects on the geometry of the vibratory mode shapes from composite laminar materials. The findings of these studies revealed that the curvature of certain mode shapes is altered due to the existence of delamination defects in the interlaminar regions of the composite laminates. In summary, it can be said that the insertion of delamination defects in composite laminar materials might cause changes in the curvature and typology of certain vibratory mode shapes, which are normally appreciated in the regions affected by the delamination phenomenon.

Despite numerous publications investigated the feasibility of the natural frequencies and mode shapes shifts as a diagnostic tool for the identification of delamination defects in composite laminar materials, the potential of the damping as a diagnostic parameter for the identification of delamination defects in fiber-reinforced polymer (FRP) panels has been scarcely addressed in the literature [26,30]. For example, S. Xing et al. [26] examined the damping changes exerted by the presence of delamination failures in composite laminates, and the findings of this study reveal that the damping of the damaged specimens is approximately 62% greater than the damping of the healthy samples. According to these literary works, the damping of composite laminates is sensitive to the presence of delamination defects indicating that the damping shifts are a good indicator of the damage conditions of FRP panels.

In comparison to the modal parameters, the application of the vibration energy as a diagnostic parameter for the identification of delamination breakages in FRP lightweight components has not been explored by the scientific community. The primary focus of this paper is to prove that the variations in overall vibration energy can be used as a simple and cost-effective approach to evaluate the presence of internal delamination defects in sandwich and monolithic composite bulkheads used in small-length FRP ships (below 50 m). For this aim, a non-destructive test is carried out to determine the overall vibration energy of bulkhead panels without delamination (known as pristine) and with delamination (known as damaged) via modal analysis. From the application point of view, this technique might be potentially used to evaluate the structural integrity of lightweight composite bulkheads in aeronautic and marine structures with the advantages of being a low-cost and straightforward approach.

The main contribution of this scientific study is to prove that the shift in the overall vibration energy of the composite specimens can be used as a simple and cost-effective approach to evaluate the structural integrity of FRP-based bulkheads used in the small-length ships. Apart from that, this study shed light on how the delamination defects affect the vibratory behavior of glass fiber epoxy composite bulkheads with and without internal defects, with a special emphasis on the damping and vibration mode shape changes. The rest of the paper is structured as follows. In Section 2, the material parameters and configuration of the intact and damaged composite bulkheads tested in this experimental campaign are introduced. Furthermore, this section gives some insight into the fabrication process used to fabricate the composite FRP bulkhead panels with and without internal delamination. Section 3 describes the modal analysis test used to determine the overall vibration energy and modal parameters of the intact and damaged composite bulkheads with different delamination sizes. Section 4 presents, analyzes, and discusses the main outcomes of this experimental campaign via modal analysis. Eventually, the last section offers a set of conclusions regarding the feasibility of the overall vibration energy, damping and vibration mode shapes for the detection and quantification of delamination in marine composite bulkheads.

## 2. Materials

**Figure 1:**
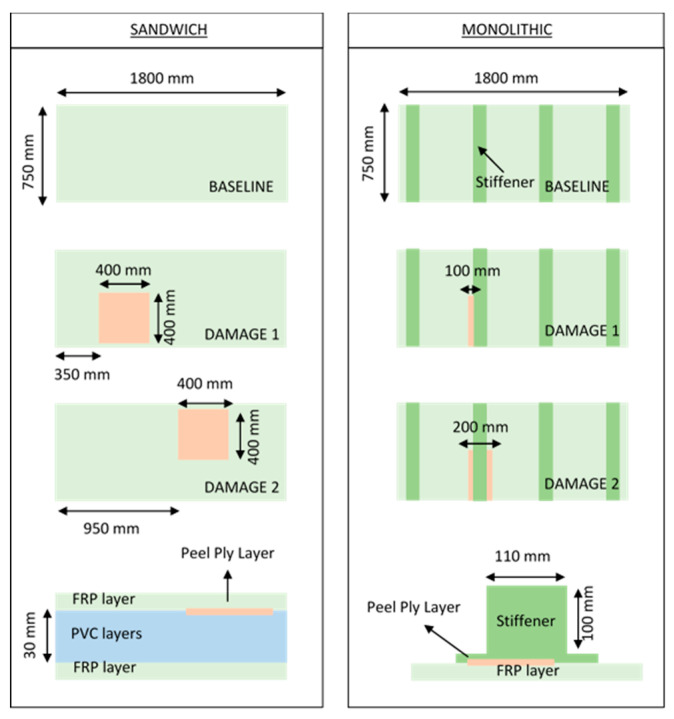
Schematic description of the six sandwich and monolithic bulkheads tested in the context of this experimental campaign. The orange square of the frontal and superior views of the panels shows the location of the delamination regions in the composite bulkheads. The dark green region in the monolithic panels displays the reinforcements of the FRP monolithic bulkheads.

Figure 1 displays a schematic description of the six sandwich and monolithic composite bulkheads tested in the context of the experimental campaign. The intact bulkheads without the presence of delamination phenomena are referred to as baseline panels while the bulkheads with delamination defects are known as damaged samples.

Three sandwich bulkhead panels with identical dimensions and three different damage levels were kindly manufactured by TUCO shipyards using the process of vacuum infusion procedure, which is a technique commonly applied for the manufacturing of composite components in the aeronautic and shipping industry [31]. The dimensions of the three composite beams are 1800 mm length, 750 mm width, and 35 mm depth. The stacking sequence of the top and lower laminate sections of the sandwich panels is [0/90, 0/90, 0/90, 0/90, 0/90]_s_ for a total number of ten layers. The core of the sandwich panels is made out of two layers of PVC with an overall thickness of approximately 15 mm.

Furthermore, three monolithic bulkhead panels with identical dimensions of 1800 × 750 × 10 mm were also fabricated using the same infusion procedure. The stacking sequence of the composite panels specified as monolithic panels is [0/90, ±45, 0/90, ±45, 0/90, ±45]_s_ with a total number of 24 layers. The stiffeners of the monolithic panels are based on six layers of glass fiber epoxy embedded in a foam core with an overall dimension of 110 × 100 mm. The stacking sequence of the stiffeners is composed of 2 layers of 600 g/m^2^ and 4 Layers of 450 g/m^2^ with ply orientations of 0/90 and +45/−45, respectively. The stiffeners of the panels are glued onto the monolithic laminate using Norpol FI-177 adhesive.

Delamination was introduced artificially by inserting a non-adhesive peel ply layer in interlaminar regions core-laminate and stiffener-laminate of the sandwich and monolithic panels, respectively. In other words, the 400 × 400 mm delamination defects of the sandwich panel are introduced in the interlaminar regions between the PVC core and the main laminate top section, as it can be seen from the frontal view of Figure 1. With respect to the monolithic panels, two different sizes of delamination with an extension of 100 × 400 mm and 200 × 400 mm were introduced in the interface between the stiffener laminate and the main monolithic beam (see frontal view of Figure 1). The location of the delamination breakages in the sandwich and monolithic composite bulkheads are represented in the orange marks shown in the panels represented in Figure 1. The core-laminate and stiffener-laminate regions of the sandwich and monolithic panels are susceptible to delamination failures owing to their low interlaminar strength.

The configuration of the sandwich and monolithic panels (baseline, damage 1, and damage 2) are shown in Figure 1. The orange square of the frontal and superior views of the panels shows the location of the delamination regions into the composite bulkheads. With respect to the sandwich panels, the same size delamination defects (400 × 400 mm) are allocated into two different locations of the panels. This practical case concentrates on the investigation of the potential of this vibration-based methodology for the localization of the delamination failures in composite bulkheads. In terms of the monolithic panels, two panels with different size defects of 100 × 400 mm and 200 × 400 mm are investigated. The fundamental purpose for this practical case is to find out if the variations of the modal parameters and overall vibration energy are suitable for the quantification of delamination damage.

The material properties of the components from the sandwich and monolithic bulkhead panels used in this research study are detailed in Table 1. The resin system used as a matrix for the composite laminates is bisphenol epoxy-based vinyl ester resin (DION IMPACT 9102-683) while the fibers used as a reinforcement in the FRP panels are based on E-Glass fibers. The bulkhead panels with the dimensions and configuration explained in the paragraph above were cured in an autoclave according to the process specifications provided by the material supplier.

## 3. Methods

### 3.1. Description of Methodology Used to Characterize the Dynamic Response of the Panels

In this paragraph, a non-destructive vibration-based methodology to identify the presence of interlaminar delamination defects in sandwich and monolithic FRP bulkheads is introduced. This strategy is based on the comparison of the vibration energy levels and modal parameters of intact and damaged composite bulkheads with different delamination scenarios.

**Figure 2:**
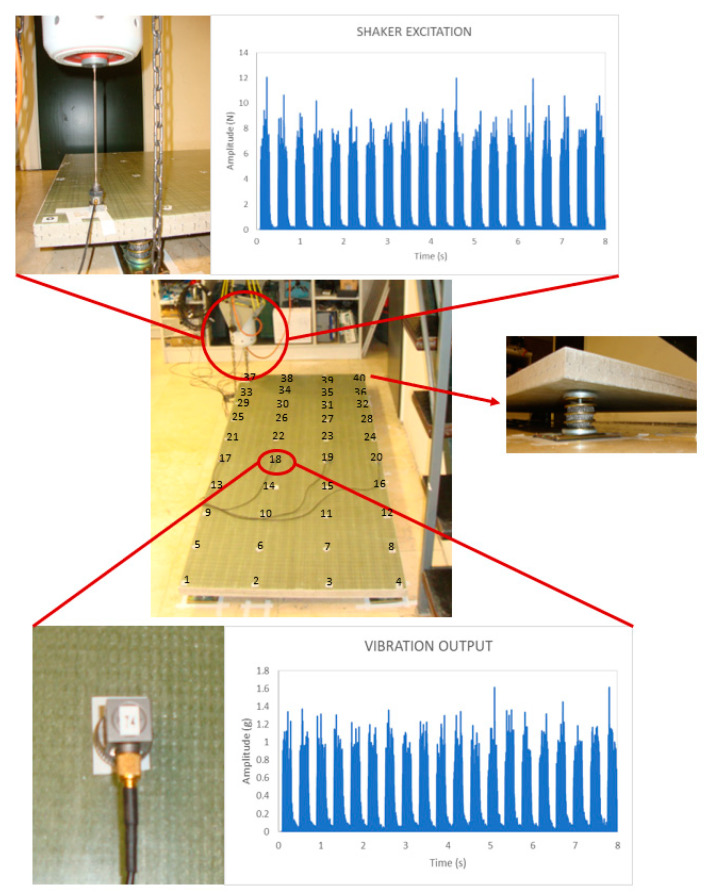
Experimental setup of the modal analysis carried out in this investigation to determine the dynamic characteristics of the sandwich and monolithic bulkheads. The shaker excitation is applied artificially through a commercial vibrometer and the vibration outputs of the panels are measured using a set of commercial accelerometers as shown in both insets of the figure.

**Figure 3:**
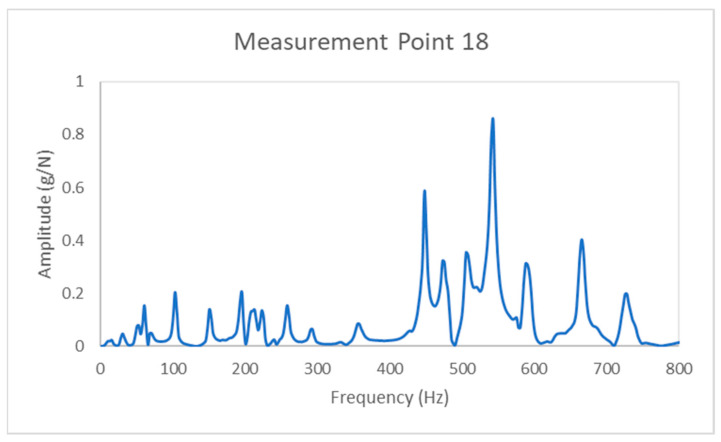
Frequency spectrum of the sandwich bulkhead panel in the measurement point 18.

The experimental setup used to extract the vibration energy and modal parameters of the composite bulkheads tested in this experimental campaign is described hereafter in Figure 2. Firstly, it was decided to allocate springs with six degrees of freedom in the four corner edges of the composite beams to support the bulkhead panels. Secondly, a vibration commercial shaker (TIRA 126/00) was used to apply periodic artificial excitations on point number 37 of the composite panels. The commercial shaker and the time domain response of the random excitations applied to the panels are shown in the upper section of Figure 2. Thirdly, the vibration signals of the composite samples are recorded for 8 s at a sampling rate of 20 kHz using an array of commercial accelerometers (Endevco, 44A16-1032). A visual description of the accelerometers and the corresponding vibratory signals recorded in the context of this experiment are shown in the bottom section of Figure 2. Last, the recorded vibration signals of the distinct composite configurations are used to obtain the FFT spectrums of the composite bulkheads with and without delamination defects as can be seen in Figure 3.

The measurement points for each specimen tested in this experimental campaign are represented on the surface of the composite panel shown in Figure 2. The overall vibration energy was measured in 40 different positions for each specimen with the aim to understand the global vibratory behavior of the intact and damaged panels. However, it should be underlined that a single measure is required to determine the overall vibration levels of the composite bulkheads, which verifies the capabilities of this methodology to carry out monitoring inspections in a cost-effective way.

The random excitation was repeated 20 times to obtain multiple realizations for each measurement point. Furthermore, the vibratory responses for each panel configuration were tested in 40 different positions along the composite panel. The standard deviation for the natural frequencies from the 40 measurements is smaller than 1%, the values of the peaks did not change, so the experimental results obtained in this experiment are rather stable and can be considered reliable.

**Figure 4:**
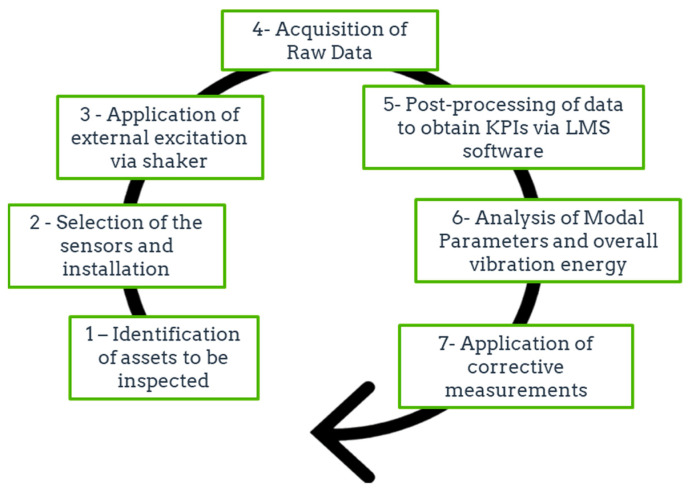
Methodology applied for the evaluation of the structural integrity of the panels.

In summary, the vibration-based method applied for the evaluation of the structural integrity of the panels is divided into seven stages that are defined in the flowchart shown in Figure 4. Initially, the maintenance team needs to identify the FRP components that are more likely to fail and install the most suitable sensors for the monitorization of the hot spots. Subsequently, a non-destructive vibration test is carried out to acquire the FFT spectrums of the panels. Eventually, the raw data are post-processed through the LMS software to obtain a set of key performance indicators (e.g., overall vibration energy) that give information regarding the damage conditions of the panels.

### 3.2. Description of Mathematical Procedures Used for Calculation of the KPI Parameters

The recorded FFT spectrums shown in Figure 3 are used to acquire the overall vibration energy and modal parameters (natural frequencies, damping, and mode shapes) of the composite bulkheads with and without delamination defects. The calculation of the above-mentioned parameters was carried out through the specialized software Siemens LMS Test 16A. The mathematical procedure applied to calculate the overall vibration energy of the panels is detailed in Section 3.2.1, while Section 3.2.2 and Section 3.2.3 are focused on the methodology used for the calculation of the damping and vibration mode shapes, respectively. A more detailed description of the theoretical background of the modal analysis can be found in reference [32].

#### 3.2.1. Determination of the Overall Vibration Energy in the FRP Bulkheads

The overall vibration energy of the panels can be defined as the sum of the vibrational energy measured within a specified frequency range. The overall vibration energy for the composite bulkheads with and without internal delamination is directly calculated from a frequency response function (see Figure 3) through Equation (1):

(1) $$RMS=\sqrt{\frac{\sum_{i=1}^{N}x_{i}^{2}}{N}}$$

where *x_i_* represents the amplitude for a certain frequency and *N* indicates the number of values analyzed in the range of frequencies. The vibration energy of the composite bulkheads was calculated in the range of frequencies between 0 Hz and 800 Hz, which is the frequency range recommended for this experimental case scenario. Therefore, an interesting aspect of this methodology is that the vibration energy levels of the composite bulkheads can be calculated easily without the need of complicated and time-consuming mathematical procedures.

Another interesting question addressed in this study deals with the number of measurement points necessary to determine the vibration energy levels of the FRP-based bulkheads. In the author’s opinion, a good approach is to measure the vibration energy in three points of the FRP bulkhead far enough away from the excitation source. As a result, only a few measurement points are necessary to evaluate the damage integrity of composite bulkheads with an area of 1800 × 750 mm in a cost-effective way.

#### 3.2.2. Determination of the Damping in the FRP Bulkheads

**Figure 5:**
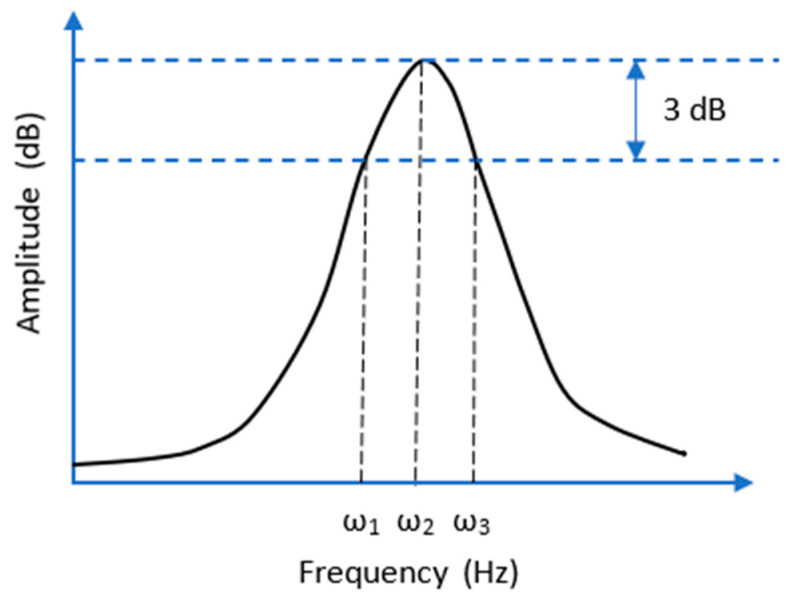
Schematic representation of the half power damping method used for the calculation of damping loss factor of the FRP bulkheads.

Damping can be defined as the energy dissipation properties of a material under cyclic stress. This parameter is an indicator of the amount of vibration energy that a material can dissipate, where higher damping values are associated with a greater capability for energy vibration dissipation. The damping of the first ten natural frequencies of the composite bulkheads is determined through the half-power method [33] through Equation (2). This parameter is calculated to evaluate the ability of intact and damaged panels to dissipate vibrational energy during the resonance phenomena.

(2) $$\zeta =\frac{\omega_{3}-\omega_{1}}{2\omega_{2}}$$

where *w*_1_, *w*_2_, and *w*_3_ are the frequencies associated with the first, second, and third points of the frequency response function (FRF) represented in Figure 5. It is relevant to note that the *w*_2_ can be defined as the frequency of the resonant peak in the bulkheads and *w*_1,3_ stands for the points of the peak located 3 dB below the maximum amplitude.

The calculation of the damping for ten frequencies of the panel was carried out using the software LMS Test Lab, which allows the simultaneous analysis of multiple measurements via the Modal Analysis module. This solution offers a powerful acquisition tool for modal parameter identification, enabling faster data interpretation and accurate measurement analysis.

#### 3.2.3. Determination of the Vibration Mode Shapes of the FRP Bulkheads

The vibration mode shapes of the first ten modes of the specimens are simulated using the modal analysis tool of the vibration analysis software Siemens LMS Test Lab. To carry out this task, the Fast Fourier Transform (FFT) spectrum characteristic for each measurement point is used to simulate the vibration mode shapes. The principal idea is to find out if the dynamic motions of the vibration mode shapes are affected by the delamination failures.

## 4. Results and Discussion

The aim of this section is to analyze the feasibility of the shifts of vibration energy and modal parameters for the detection and quantification of delamination failures in FRP-based bulkheads. The section is divided into three subsections. Section 4.1 and Section 4.2 are devoted to the investigation of the influence of delamination defects on the overall vibration energy and damping of the composite laminates. Section 4.3 analyzes the effect of the delamination defects on the vibratory mode shapes of the sandwich and monolithic bulkhead panels. The fundamental idea is to find out if the variations of these parameters can be used as a diagnostic tool to evaluate the damage conditions of the bulkheads used in small-scale ships.

### 4.1. Effect of Delamination on the Overall Vibration Energy of Composite Bulkheads

This section looks at the influence of delamination defects on the vibration energy levels of composite bulkheads used in marine applications. To address this investigation, the levels of vibration energy for the identical sandwich and monolithic bulkheads with three different damage states are compared in order to find out how the delamination breakages produce changes in the overall vibration energy of the materials.

**Figure 6:**
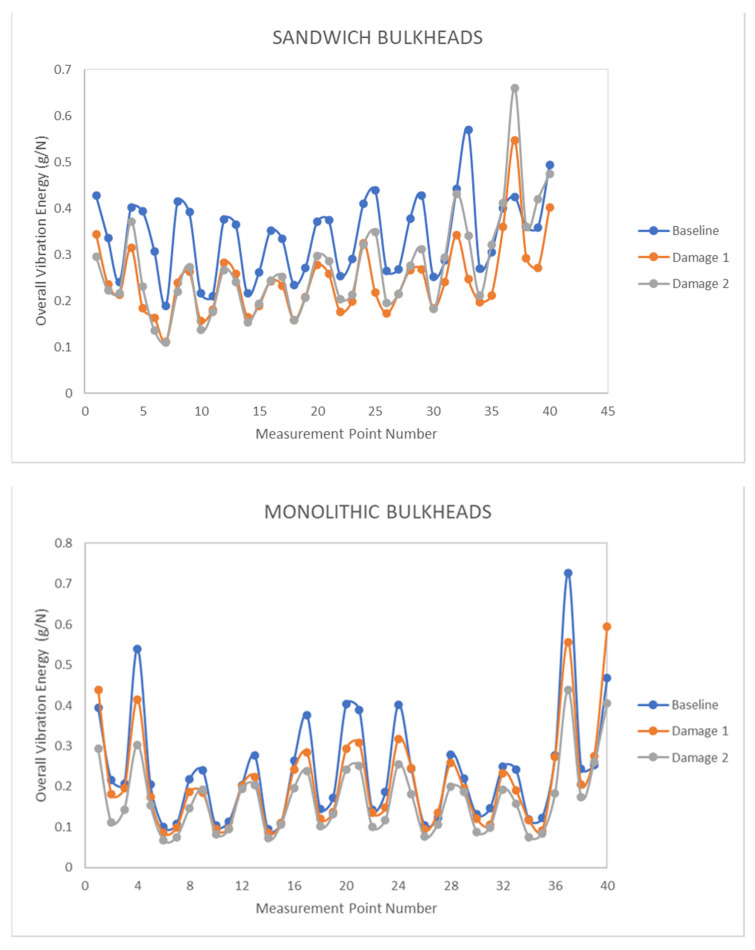
Overall vibration energy of the sandwich/monolithic bulkheads for each measurement point.

Figure 6 displays the amount of vibration energy in the sandwich and monolithic bulkheads for each of the 40 measurement points of the panels. The blue line shows the overall vibrational energy levels of the intact composite panels referred to as baseline while the orange/grey curves are related to the overall vibration energy of the panels with delamination breakages known as damaged specimens. In general, it is observed that the overall vibration energy levels for the pristine bulkheads are greater than the overall vibration energy of the damaged panels, which indicates that there is a reduction of vibratory energy due to the presence of the delamination defects. This reduction of vibratory energy can be explained by the energy dissipation phenomena generated by multiple frictions between the upper and lower surfaces of the delamination defects at certain frequencies. Similar dynamic behavior is appreciated from the signals recorded in the monolithic panels. The overall vibration energies of the intact and damaged sandwich/monolithic bulkheads can also be found in Table A1 and Table A2 of Appendix A.

**Figure 7:**
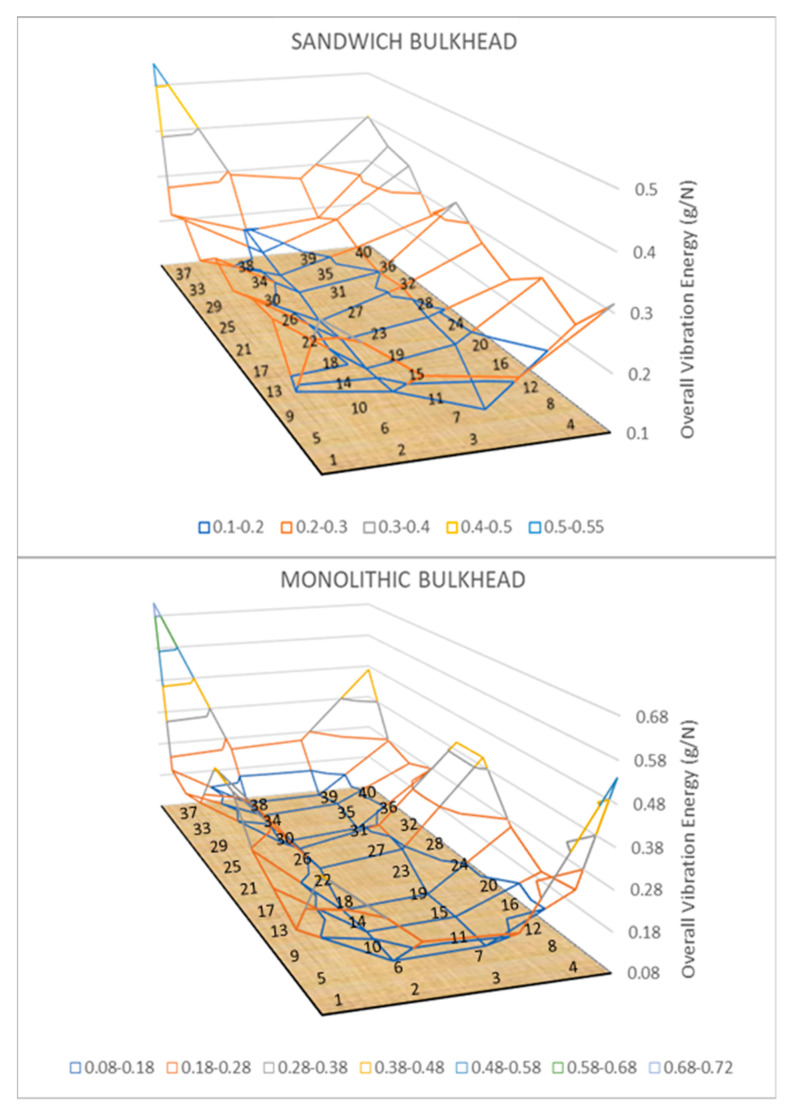
Three-dimensional map of vibratory energy levels in sandwich and monolithic panels.

Figure 7 reveals a three-dimensional diagram of the vibration energy levels of the sandwich and monolithic bulkheads. The graph shows the overall vibration energies recorded in the forty measurement points over the surface of the composite panel bulkheads. For the sandwich bulkhead, three zones with distinct energy levels in the FRP panels (central, lateral, and edges) can be clearly distinguished. In the central part of the specimens, small vibration energies in the range from 0.1 to 0.2 g/N are recorded by the accelerometers. The lateral edges of the panel have vibratory energies in the range from 0.2 to 0.35 g/N, which is a good indicator of the strong lateral vibrations of the bulkhead. The edges of the panel show the highest energy values for the panel (0.35 and 0.55 g/N) due to the application of the shaker excitation in point 37, which corresponds with the maximum of vibratory energy. Similar behavior in terms of overall vibration energy is reported for the monolithic panels illustrated in the bottom part of Figure 7.

**Figure 8:**
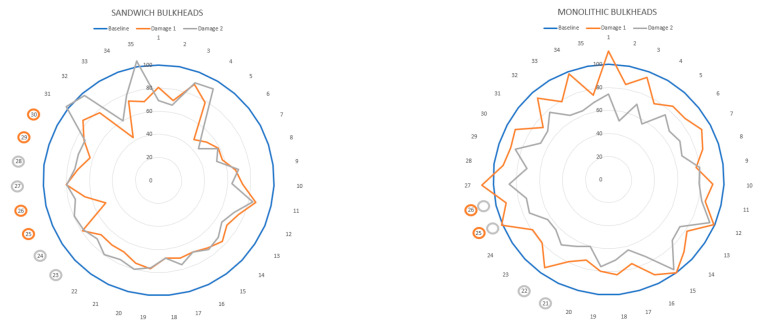
Vibrational energy of the sandwich and monolithic bulkheads in percentage value as a function of the measurement point.

The energy variations in percentage values for the sandwich/monolithic panels due to delamination defects are represented in Figure 8. The vertical axis of the radial graph shows the energy levels of the panels in percentage values, where the blue curve represents the energy of the neat specimen and the orange/grey curves refer to the energy levels of the panels with delamination defects. The circular axis of the graphic represents the measurement point of the panel. It is worthy to note that the measurement points 36–40 are not included in this analysis because the regions around the vibration shaker are not optimum for this type of analysis due to the uncertainties generated by the excitation source.

Figure 8 presents an interesting visual proof of the significant percentage of decay of the overall vibration energy due to the inclusion of delamination defects in the composite bulkheads, which is attributed to energy dissipation phenomena generated by the delamination defects. The vibration measures taken on the composite regions with delamination defects have been highlighted with circles on the circular axis of the radar graphs. The graph shows that the measurement point numbers 23 to 30 are associated with the delamination regions of the sandwich panels, while the measurement points (21, 22, 25, and 26) correspond to the regions of the monolithic panel with delamination failures.

The findings of this study support the idea that the overall vibration energy is a good key performance indicator for the detection and quantification of delamination defects. For example, it is observed that the overall vibration energy of the monolithic bulkheads decreases approximately 10% for the panel damaged with a delamination size of 100 × 400 mm and around 25% in the composite panel with the defect of 200 × 400 mm. From the authors’ point of view, the higher percentual decrement of vibration energy in the panel with the 200 × 400 mm delamination failure is induced by the larger size of the defect that increases the amount of energy dissipated. Thus, it can be concluded that delamination defects produce phenomena of energy dissipation that result in a decay of overall vibration energy, which is proportional to the size of the delamination defect.

The feasibility of the overall vibration energy as a key performance indicator for the localization of delamination defects in composite laminates is not evident from the experimental data provided by this research study. The overall vibration energy of the two sandwich panels with identical delamination defects of 400 × 400 mm decreases by more than 20% in the majority of the measurement points. However, the shift of vibration energy recorded in the points around the delaminated regions of the bulkhead panels is similar to that of the areas in good conditions. In the author’s opinion, this behavior might be explained because the intensity of the energy dissipation phenomena generated by the frictions of the delaminated layers are barely affected by the defect localization. Thus, the overall vibration energy variations are not sufficient for the localization of delamination defects in the sandwich/monolithic panels.

In spite of the fact that a large number of publications are focused on the variation of the modal parameters, the potential of the overall vibration energy for the detection of delamination defects in composite laminar materials has been not explored in the literature. Indeed, the majority of the research publications published in the field of vibration-based structural health monitoring (VSHM) are concentrated on the variation of the modal parameters. A couple of interesting contributions to the field were reported by K. Alnefaie et al. [24] and M. Imran [29]. The first one [24] carried out a detailed analysis of the natural frequency changes in composite laminates due to internal delamination defects. From the results, a reduction of the value of the natural frequencies in the laminates is highlighted due to the presence of delamination defects in the laminates. However, the variations appreciated on the natural frequencies of the composite laminates with internal delamination are marginal (less than 2%) as these changes are within the range of precision of the experiments. The second one [29] investigated the effect of the delamination on the vibratory mode shapes of carbon fiber reinforced polymer composites. From the results of this numerical study, it can be seen that the insertion of delamination defects in the laminates affected the curvature of certain vibratory mode shapes of the laminate.

On the basis of these results, it is noticed that the changes in overall vibration energy show great potential for the detection of delamination defects in sandwich and monolithic composite bulkheads. Therefore, the results of this paper can be used as a proof-of-concept that the changes of vibration energy can be used as a diagnostic parameter for the detection of delamination defects in composite bulkheads. From the authors point of view, this technology has important implications for the detection of defects in a wide range of composite applications such as wind energy turbines, bulkheads, aircraft structures, etc. In any case, it is important to be cautious because these interesting results for the detection of delamination in composite bulkheads were obtained on a lab-scale and, therefore, the feasibility of this methodology still needs to be proven in an industrial case scenario with sea waves impacts, propeller excitations, and other external excitations that may disturb the vibration measurements.

### 4.2. Effect of the Delamination on the Damping of Composite Bulkheads

This paragraph analyzes how the delamination defects affect to the damping behavior of the composite panels. To carry out this investigation, the damping for three identical sandwich/monolithic composite bulkheads with different damage states are compared in order to evaluate the influence of this type of defects on this key performance indicator. The damping values of the first ten vibration modes of the sandwich and monolithic bulkheads with and without delamination defects are compared in Table 2 and Table 3, respectively.

The experimental data given in Table 2 reveals that the changes of the damping are not the same for the different modes. A significant damping increase of approximately 100% was observed for Modes 5 and 8 of the sandwich panels. Meanwhile, the changes of the damping for Modes 1 and 2 are relatively small (around 10%). Therefore, it can be said that some modes are more sensitive to fluctuate due to the presence of delamination failures. In general, it can be observed that the changes of the damping are substantial for the first 10 modes with variations of up to 200% for some of the high-frequency modes.

The damping percentual changes of the sandwich panels (see Table 2) are quite dependent on the vibration mode shape and, therefore, not all the modes show the same tendency. On one side, it is appreciated that there is a substantial damping increase of more than 200% in the ninth and tenth vibration mode shape, which indicates the existence of intense energy dissipation phenomena caused by the interfacial friction between the adjacent layers of the delamination defects. On the other side, important percentual damping decrements are reported for certain mode shapes. Therefore, it can be concluded that the damping percentual changes are quite dependent on the type of vibration mode shape. Similar results are reported for the pristine and damaged monolithic panels as detailed in Table 3, which provides the damping for the ten first vibration modes of the monolithic bulkheads with and without delamination.

The findings of this paper are in line with the results reported in other research works published in the literature. For example, Z. Kiral et al. [30] carried out a comparative study on the damping of intact and delaminated composite panels impacted at three different levels of energies, 10 J, 15 J, and 20 J. The findings of this study showed that the damping ratio of the composite laminates is sensitive to the presence of delamination defects that cause relevant changes in the magnitude of this modal parameter. In parallel, other authors such as S. Xing et al. [26] also investigated the changes in the damping ratio due to the presence of delamination defects in composite laminates. The results of this paper reveal that the damping ratio of the composite specimens increases from 1.05 to 1.71 due to the presence of delamination failures. According to the results given in the literature, the effect of the delamination on the natural frequencies of glass fiber epoxy specimens is modest. On the contrary, the damping of the composite laminates is more sensitive to the delamination presence indicating that the variations of the damping seem to be a better indicator of delamination damage.

**Figure 9:**
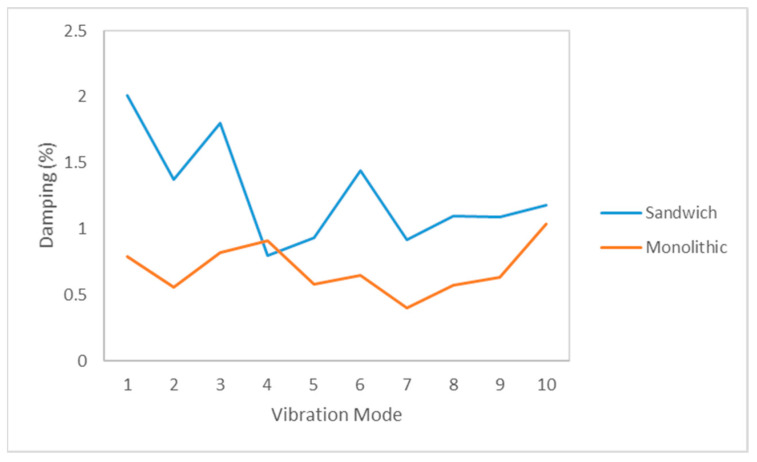
Comparison of the damping ratio in the intact sandwich and monolithic panels tested in this investigation.

It is well known the heterogeneous structure of the composite materials facilitates the phenomena of dissipation of energy due to their multiple numbers of layers and anisotropic structure. Figure 9 shows a comparison of the damping values in the pristine sandwich and monolithic panels for the 10 first modes. The graph reveals that the damping values of the sandwich panels are higher than the damping of the monolithic panels, which indicates a greater capability of this material for the dissipation of energy. In comparison with the monolithic panels, the sandwich panel presents two intermediate PVC layers that produce additional phenomena of dissipation of energy, and lead to a higher damping ratio in the majority of the modes. Therefore, it can be concluded that the sandwich bulkheads dissipate more vibratory energy than the monolithic panels, and therefore the selection of these types of composite materials is more appropriate for applications where vibrations are a source of problems.

In conclusion, it can be said that the delamination defects tend to increase the damping because the delamination regions act as dampers of the laminates. However, these changes are quite dependent on the vibration mode as the ability of the frequencies to excite a particular defect is related to their wavelength. In the authors’ view, the changes of damping are an interesting parameter but are not recommended as a diagnostic tool for the detection of delamination defects in composite bulkheads because they are strongly dependent on the frequency mode shape and do not give consistent results for all the modes.

### 4.3. Effect of the Delamination on the Vibration Mode Shapes of Composite Bulkheads

Another diagnostic tool analyzed in this paper is based on tracking the local changes of the vibration mode shapes due to the presence of delamination defects in the composite panels. This strategy is based on the analysis of the relative displacements that the delamination defects exert on the vibration mode shapes of composite bulkheads. Therefore, the primary objective of this section is to identify the local changes, which may appear in certain mode shapes of the laminates due to the presence of delamination failures.

**Figure 10:**
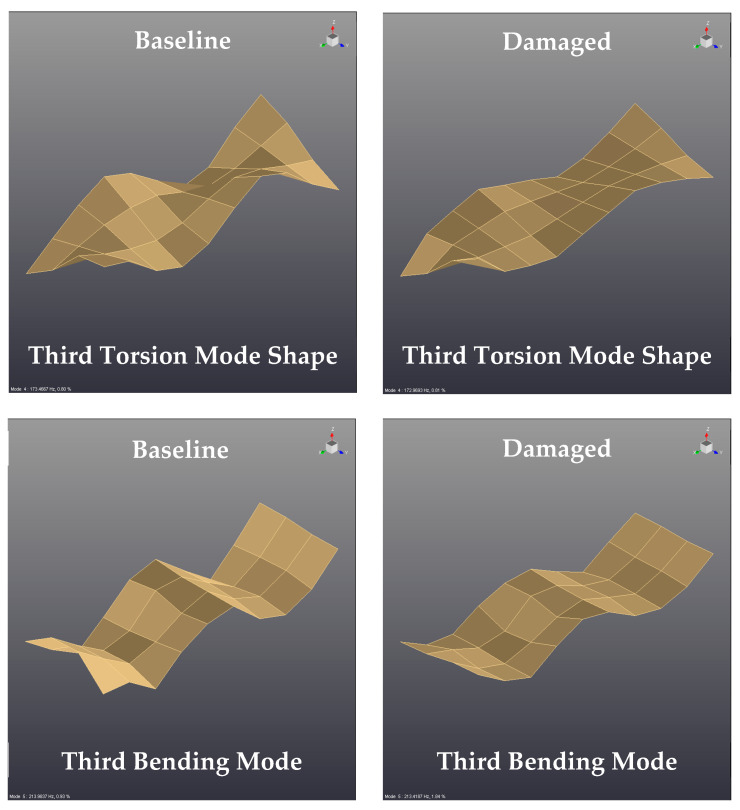
Comparison of vibration mode shapes for sandwich panels in intact and damage conditions.

Modes 4 and 5 of the sandwich panels with and without internal delamination are represented in Figure 10, which correspond to the third torsion and bending mode shape of the sandwich bulkheads. In general, it is observed that the geometry of the vibration mode shapes is not affected by the delamination defects as the type of mode shape (third torsion and bending mode shape) remains constant for the pristine and damaged bulkheads. However, it is interesting to note that the relative deformations of the mode shapes are smaller for the damaged panels as can be deducted from Supplementary Videos S1–S4. This behavior might be explained due to the loss of vibratory energy caused by the dissipation energy phenomena generated by the delamination defects.

**Figure 11:**
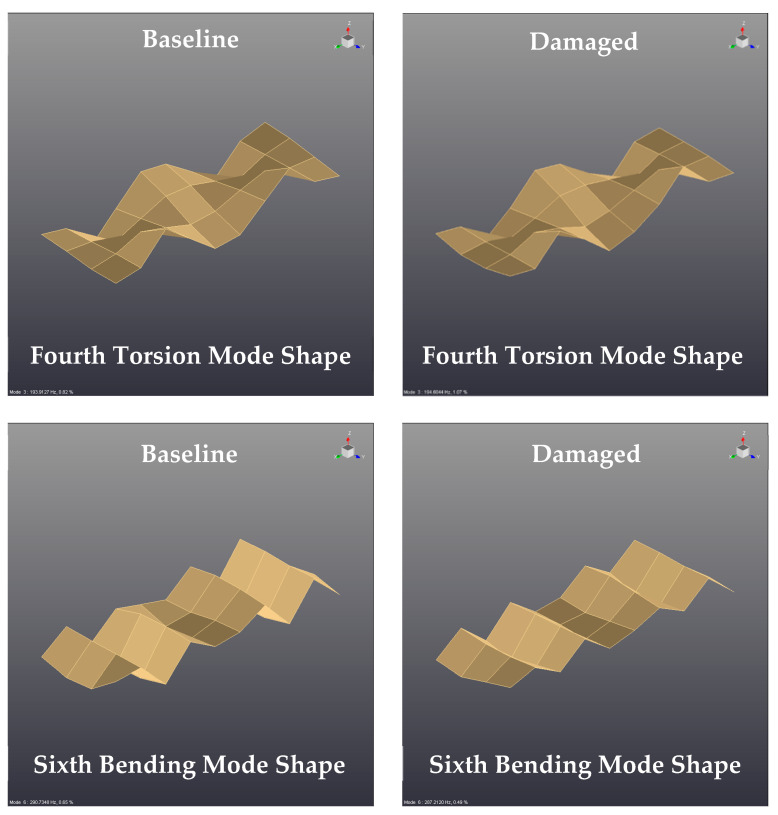
Comparison of the vibration mode shapes for monolithic panels in intact and damaged conditions.

Modes 4 and 5 of the monolithic panels with and without internal delamination are represented in Figure 11. These vibration mode shapes correspond to the fourth torsion and sixth bending mode shape of the monolithic bulkheads. The typology of the vibration mode shape can be easily deducted from the images displayed in this figure. As noted, the type of vibration mode shape remains constant for the intact and damaged bulkheads. The videos of the vibration mode shapes illustrated in this figure are given in Supplementary Videos S5–S8. In this particular case, significant changes in the relative deformations of the modes have not been observed.

The influence of delamination defects in the vibration mode shapes of composite laminates has been investigated previously by other researchers [27,28,29]. A very interesting study was reported by the authors of [29], which investigated the effect of the delamination on the vibratory mode shapes of carbon-fiber-reinforced polymer composites. From the results of this numerical study, it can be seen that the insertion of delamination defects in the laminates affected the geometry of certain vibratory mode shapes of the laminate.

In summary, the findings of the current study reported that there are small deviations in the relative displacements of some mode shapes, which might be induced by the dissipation phenomena generated by the delamination defects of the bulkheads. However, the authors of this paper discarded the variation of the mode shapes as a potential tool for the detection of delamination failures due to the large number of measurement points required for the calculation of the modes (40 measurement points per sample in this experimental campaign) and the long time required to post-process and analyze the experimental data.

## 5. Conclusions

This paper has considered the problem for the identification, quantification, and localization of delamination defects in marine composite bulkheads, which are used for the design and construction of small-length ships (less than 500 GT). To evaluate the integrity of the panels, the overall vibration energy, damping and vibration mode shapes of six sandwich and monolithic composite panels with different levels of damage are considered. On the basis of the experimental results, the following conclusions may be drawn.

The variation of vibration energy in composites with and without internal delamination is considered as the best key performance indicator. This study proves that the overall vibration energy of laminates with delamination is inferior with respect to the neat specimens. This can be explained due to the fact that composites with delamination defects dissipate higher energy due to multiple interlaminar frictions among the damaged layers. As a result, it is concluded that the shift of vibration energy is a reliable and economic parameter for the detection of delamination in composite bulkhead panels.

In comparison to the natural frequencies, the damping of the damaged composite specimens is more sensitive to the presence of delamination phenomena. In general, the delamination defects tend to increase the damping of the composite bulkheads because the delamination regions act as dampers. However, these changes are quite dependent on the vibration mode as the ability of the frequencies to excite the defect is related to their wavelength. Additionally, the paper reveals that the sandwich bulkheads dissipate more vibratory energy than the monolithic panels, and therefore the selection of these type of composite materials is more appropriated for applications where vibrations are a source of problems.

Apart from that, this paper investigates the changes of the vibration mode shapes due to the incorporation of delamination regions in composite laminates. The findings of this research highlighted that the relative displacements of certain vibration mode shapes are slightly affected by the delamination failures. However, the authors of this paper discarded the variation of the mode shapes as a potential tool for the detection of delamination failures because the reported displacement changes are small for the modes investigates in this research study.

In summary, this paper introduces an innovative non-destructive method for the detection and characterization of internal delamination defects in composite panels based on the changes of the overall vibration energy. Since vibration measurements can be easily acquired, this approach provides a cost-effective approach to identify the existence of delamination defects in composite laminar materials. It is important to highlight that it is only required one single measurement point to calculate the overall vibration energy of panels with an area of 1800 × 750 mm (in this case, a vessel bulkhead), and therefore it is not necessary to carry out a large experimental testing campaign to evaluate the damage integrity of the bulkhead panels. The findings of this research possess important industrial applications for monitoring destructive delamination failures in the FRP-based bulkheads used in aircrafts, wind turbines, vessels, and other engineering composite structures in which this approach is an excellent structural assessment tool and an evident advantage for their extension of life-cycle.

## Figures and Tables

**Table 1:** Material properties of the sandwich and monolithic composite laminates tested.

	Sandwich Panel	Monolithic Panel	Both Panels	
Description	PVC Core	Foam	Resin	E-Glass
Density (kg/m^3^)	80 (ISO 845)	48 (ISO 845)	1010 (ISO 2811-2001)	2620
Tensile Modulus (MPa)	95 (ASTM D 1623)	55 (ASTM D 1623)	3400 (ISO 527-1993)	72400 MPa
Tensile Strength (MPa)	2.5 (ASTM D 1623)	1.4 (ASTM D 1623)	79 (ISO 527-1993)	3450 MPa

**Table 2:** Damping values for the pristine and damaged sandwich panels. The variation of the damping due to the presence of delamination defects is calculated through the damping percentage deviation in intact and damaged composite panels.

Mode	Baseline (%)	Damage 1 (%)	Damage 2 (%)	Variation 1 (%)	Variation 2 (%)
1	2.01	2.26	1.77	+12.44	−11.94
2	1.37	1.51	1.52	+10.22	+10.95
3	1.80	1.01	2.09	−43.89	+16.11
4	0.80	0.81	0.90	+1.25	+12.50
5	0.93	1.84	1.90	+97.85	+104.30
6	1.44	1.50	1.75	+4.17	+21.53
7	0.92	0.45	1.22	−51.09	+32.61
8	1.10	2.72	2.45	+147.27	+122.73
9	1.09	1.57	3.28	+44.04	+200.92
10	1.18	1.14	3.87	−3.39	+227.97

**Table 3:** Damping values of intact and damaged monolithic panels. The variation of the damping due to the presence of delamination defects is calculated through the damping percentage deviation in intact and damaged composite panels.

Mode	Baseline (%)	Damage 1 (%)	Damage 2 (%)	Variation 1 (%)	Variation 2 (%)
1	0.79	1.15	0.92	+45.57	+16.46
2	0.56	0.72	0.63	+28.57	+12.50
3	0.82	1.07	0.90	+30.49	+9.76
4	0.91	0.76	0.66	−16.48	−27.47
5	0.58	0.50	0.49	−13.79	−15.52
6	0.65	0.49	0.54	−24.62	−16.92
7	0.40	0.53	0.52	+32.50	+30.00
8	0.57	0.78	1.12	+36.84	+96.49
9	0.63	0.81	0.73	+28.57	+15.87
10	0.93	0.83	0.65	−10.75	−30.11

## Data Availability

Data available in Appendix A and Supplementary Materials Section.

## Supplementary Materials

The following are available online at https://www.mdpi.com/article/10.3390/s21082843/s1, Video S1, Video S2, Video S3, Video S4, Video S5, Video S6, Video S7, Video S8.

## Appendix A

**Table A1:** Overall vibration energy of the sandwich bulkheads for each measurement point. The table also includes the variation of the vibration energy due to the delamination defects.

	Overall Vibration Energy (g/N)				
Point	BL	D1	D2	Variation 1	Variation 2
1	0.426905827	0.344146271	0.294786214	19%	31%
2	0.335224636	0.236063436	0.222123942	30%	34%
3	0.241102549	0.213642882	0.216918313	11%	10%
4	0.401735692	0.314916327	0.3712156	22%	8%
5	0.393031651	0.183751761	0.230269725	53%	41%
6	0.306916827	0.163472124	0.135799769	47%	56%
7	0.189800935	0.111700993	0.110267512	41%	42%
8	0.414044973	0.239602475	0.219289441	42%	47%
9	0.391480561	0.262876858	0.273557722	33%	30%
10	0.216131847	0.157195121	0.137943697	27%	36%
11	0.209673014	0.18107532	0.175630913	14%	16%
12	0.376038709	0.283049929	0.266659787	25%	29%
13	0.364771818	0.258298157	0.239880167	29%	34%
14	0.215663846	0.165195196	0.153858675	23%	29%
15	0.261067341	0.188927422	0.19332051	28%	26%
16	0.351846897	0.242475634	0.243196265	31%	31%
17	0.333587537	0.233169104	0.252479827	30%	24%
18	0.234940496	0.158827466	0.159177224	32%	32%
19	0.271268942	0.208251795	0.206363335	23%	24%
20	0.370800866	0.276957526	0.297143859	25%	20%
21	0.374304724	0.258190244	0.28528846	31%	24%
22	0.254272851	0.175505256	0.202890063	31%	20%
23	0.290559045	0.199447751	0.213595514	31%	26%
24	0.410513468	0.325156839	0.320471512	21%	22%
25	0.439011918	0.218286301	0.348212327	50%	21%
26	0.264407272	0.172424359	0.195428956	35%	26%
27	0.268820974	0.214931131	0.214282923	20%	20%
28	0.377050184	0.26585797	0.276152229	29%	27%
29	0.427998801	0.267415169	0.312002169	38%	27%
30	0.251774497	0.184272954	0.182382097	27%	28%
31	0.287910527	0.240782076	0.293984671	16%	−2%
32	0.441554107	0.343019273	0.430902545	22%	2%
33	0.568963811	0.246610249	0.340722929	57%	40%
34	0.268896253	0.197522599	0.210868623	27%	22%
35	0.305720593	0.212063684	0.321152195	31%	−5%
36	0.400781874	0.359196857	0.411597175	10%	−3%
37	0.424655096	0.546752086	0.659485895	−29%	−55%
38	0.359215711	0.29221955	0.359351511	19%	0%
39	0.358488464	0.270811848	0.419124189	24%	−17%
40	0.494439681	0.402153815	0.474041485	19%	4%

**Table A2:** Overall vibration energy of the monolithic bulkheads for each measurement point. The table also includes the variation of the vibration energy due to the delamination defects.

	Overall Vibration Energy (g/N)				
Point	BL	D1	D2	Variation 1	Variation 2
1	0.394132899	0.438939105	0.292753438	−11%	26%
2	0.215853961	0.181011162	0.111274186	16%	48%
3	0.206609793	0.195465194	0.143791587	5%	30%
4	0.540248077	0.414939294	0.303449511	23%	44%
5	0.205780318	0.173723343	0.153202791	16%	26%
6	0.100663082	0.085509929	0.06777631	15%	33%
7	0.107173445	0.09822852	0.075142056	8%	30%
8	0.217791131	0.186497605	0.145686263	14%	33%
9	0.241075739	0.184917188	0.191823916	23%	20%
10	0.10445224	0.094578558	0.081978228	9%	22%
11	0.113847605	0.097910572	0.094374666	14%	17%
12	0.20332978	0.202006733	0.19441702	1%	4%
13	0.276222215	0.224239327	0.203896088	19%	26%
14	0.094556748	0.085682833	0.072356743	9%	23%
15	0.109721725	0.109583563	0.105939862	0%	3%
16	0.264778002	0.242471547	0.196427686	8%	26%
17	0.376004331	0.283937575	0.237972278	24%	37%
18	0.145516684	0.120465895	0.101887149	17%	30%
19	0.173069721	0.138221541	0.131471964	20%	24%
20	0.404324992	0.293334474	0.242535261	27%	40%
21	0.389805084	0.30729153	0.250827443	21%	36%
22	0.142724913	0.13476233	0.099783871	6%	30%
23	0.186421837	0.148288012	0.117399374	20%	37%
24	0.402310347	0.317151035	0.254757197	21%	37%
25	0.243591316	0.245203987	0.181151325	−1%	26%
26	0.105209315	0.09556496	0.076774235	9%	27%
27	0.12295233	0.134900598	0.106224494	−10%	14%
28	0.279312076	0.257782202	0.1992603	8%	29%
29	0.219901424	0.196050817	0.186562446	11%	15%
30	0.131278963	0.120731761	0.087894905	8%	33%
31	0.145675932	0.105988054	0.098200329	27%	33%
32	0.249603817	0.233742684	0.192714061	6%	23%
33	0.242647775	0.19106612	0.157792748	21%	35%
34	0.118588052	0.117159551	0.075505717	1%	36%
35	0.122531773	0.091277858	0.083380367	26%	32%
36	0.276902934	0.273313941	0.1833281	1%	34%
37	0.727643162	0.556901319	0.438377194	23%	40%
38	0.244268715	0.20463043	0.174603911	16%	29%
39	0.253921738	0.275020759	0.261055755	−8%	−3%
40	0.468427294	0.595396127	0.405006692	−27%	14%
